# Interrater and Intrarater Reliability of the Tuck Jump Assessment by Health Professionals of Varied Educational Backgrounds

**DOI:** 10.1155/2013/483503

**Published:** 2013-12-16

**Authors:** Lisa A. Dudley, Craig A. Smith, Brandon K. Olson, Nicole J. Chimera, Brian Schmitz, Meghan Warren

**Affiliations:** ^1^North Country HealthCare, 301 South 7th Street, Williams, AZ 86046, USA; ^2^Proactive Physical Therapy, 3945 East Paradise Falls Drive No. 109 Tucson, AZ 85712, USA; ^3^Department of Physical Therapy and Athletic Training, Northern Arizona University, P.O. Box 15105, Flagstaff, AZ 86011, USA; ^4^Athletic Training Department, Daemen College, 4380 Main Street, Amherst, NY 14226-3592, USA; ^5^DeRosa Physical Therapy, 1301 West University Avenue, Flagstaff, AZ 86001, USA

## Abstract

*Objective*. The Tuck Jump Assessment (TJA), a clinical plyometric assessment, identifies 10 jumping and landing technique flaws. The study objective was to investigate TJA interrater and intrarater reliability with raters of different educational and clinical backgrounds. *Methods*. 40 participants were video recorded performing the TJA using published protocol and instructions. Five raters of varied educational and clinical backgrounds scored the TJA. Each score of the 10 technique flaws was summed for the total TJA score. Approximately one month later, 3 raters scored the videos again. Intraclass correlation coefficients determined interrater (5 and 3 raters for first and second session, resp.) and intrarater (3 raters) reliability. *Results*. Interrater reliability with 5 raters was poor (ICC = 0.47; 95% confidence intervals (CI) 0.33–0.62). Interrater reliability between 3 raters who completed 2 scoring sessions improved from 0.52 (95% CI 0.35–0.68) for session one to 0.69 (95% CI 0.55–0.81) for session two. Intrarater reliability was poor to moderate, ranging from 0.44 (95% CI 0.22–0.68) to 0.72 (95% CI 0.55–0.84). *Conclusion*. Published protocol and training of raters were insufficient to allow consistent TJA scoring. There may be a learned effect with the TJA since interrater reliability improved with repetition. TJA instructions and training should be modified and enhanced before clinical implementation.

## 1. Introduction

Annually, 80,000 anterior cruciate ligament (ACL) injuries occur in the United States with an estimated cost of almost a billion dollars [1]. The most common mechanisms of ACL injury are noncontact in nature, characterized by sudden deceleration prior to a landing motion or change of direction [2, 3]. These noncontact injuries may be due to coordination failure involving a complete and momentary loss of normal protective muscle support [4]. People at high risk of ACL injury frequently demonstrate high dynamic knee valgus (i.e., knee abduction moment) during landing from jumping, which may be due to decreased neuromuscular control [3, 5]. Current literature has proposed several laboratory-based tools to identify risk factors for ACL injury [6]. However, these screening tools require expensive 3D motion capture equipment, highly trained staff, and significant amount of time to administer and analyze rendering these tools inefficient and impractical for a clinical setting [6].

Several jumping and landing tests are used in the clinical setting, including the landing error scoring system (LESS), the drop jump video screening test, and the tuck jump assessment (TJA) [6–9]. The TJA may offer clinical advantages over the other tests. For example, the TJA protocol, unlike the other two tests, starts and stops from ground level instead of jumping from a box; this better represents techniques encountered in normal jumping activities [6, 8]. The TJA protocol also requires participants to jump for 10 seconds, while the LESS and drop jump video screening test require only 1 to 2 jumps [6–9]. Therefore, the TJA evaluates a measure of performance endurance, introducing a potential fatigue effect that might highlight landing flaws not observable in 1 to 2 jumps [6–9]. The TJA was developed as a practical “clinician friendly” plyometric assessment, identifying jumping and landing technique flaws pertaining to risk of ACL injury [8, 10]. Although there are no published reports of the TJA being widely used, the availability of test protocol and minimal equipment required may make this a favored tool to use in varied clinical settings with diverse personnel. The TJA includes 10 technique flaws related to jumping and landing that are scored qualitatively as either having the flaw or not [6, 8]. Empirical evidence suggests a participant who demonstrates greater than or equal to 6 out of 10 technique flaws during the TJA should be targeted for intervention to address flaws, such as correcting lower extremity valgus at landing [6, 8, 10]. The TJA requires minimal equipment (e.g., video cameras and tape markers) and takes only several minutes to administer. Scoring follows standard criteria for each technique flaw and can be completed relatively quickly by watching video playback. These features make the TJA a practical screening tool for injury risk assessment in a clinical setting for people of different educational backgrounds and levels of experience, as it is currently being used.

Previous literature reported the interrater reliability of the TJA as high with percentage exact agreement (PEA) of two testers across all scoring criteria of 93% (range 80%–100%) when scoring 10 participants [11]. The same study also reported intrarater reliability to be high (PEA of 96%–100%) [11]. Although reliability was reported high, several limitations in study design necessitate further study of reliability of the TJA. Specific information concerning training and background of TJA scoring for the raters was not included, limiting generalizability to clinicians unfamiliar with jumping assessment. Additionally, the small sample size may have allowed raters to remember previous scores, introducing bias, resulting in higher intrarater reliability.

Reliability has not been tested with raters of different educational backgrounds or levels of experience. Demonstrating that scoring is consistent between raters of different educational and experiential backgrounds would allow implementation of the TJA in the clinical and performance settings with improved understanding of accuracy.

The purpose of this study was to investigate intrarater and interrater reliability of the TJA with raters of different educational backgrounds and levels of clinical experience with healthy injury-free men and women. The hypothesis for this study was that the raters would demonstrate good intra- and interrater reliability for the TJA and that there would be no difference in TJA total score between raters of different educational and/or experiential backgrounds.

## 2. Methods

### 2.1. Participants

A sample of 108, both undergraduate and graduate, recreationally active students who were not currently involved in college athletics, were recruited for participation. All participants were healthy, injury-free men and women, 18 to 24 years old, without prior tuck jump training. From this cohort, the videos of 40 participants (*n* = 13 men and *n* = 27 women) were randomly selected for this reliability study. Participants received a full explanation of the nature, purpose, and risks of the study and were given the opportunity to ask questions. The Physical Activity Readiness Questionnaire (PAR-Q) was administered to screen for contraindications for testing [12]. Participants with any positive responses were excluded. All participants signed an informed consent document approved by the Institutional Review Board at Northern Arizona University before participating in the study.

### 2.2. Tuck Jump Assessment

Prior to participants completing the TJA, height and weight were measured with a wall-mounted stadiometer and digital scale (Cardinal Scale, Webb City, MO, USA).

**Figure 1:**
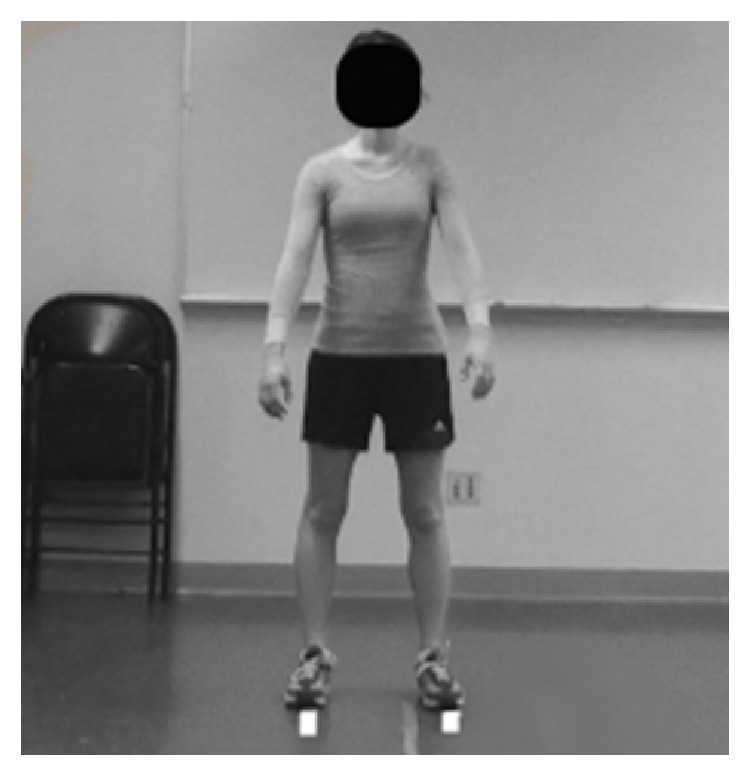
Tuck jump assessment starting position.

The TJA was performed using instructions from a previously published TJA study by the developers of the test [8]. Initial set up for the TJA required 2 two-dimensional video cameras (Sony Handycam, Sony Corporation, San Diego, CA and JVC camcorder JVC Americas Corporation, Wayne NJ) on tripods to provide sagittal and frontal views of the participants. Two pieces of masking tape were placed on the ground, parallel to each other 8 inches apart. Participants were instructed to stand with one foot on each tape strip to ensure proper positioning for the cameras (Figure 1). The participants were instructed in purpose and protocol of TJA test which included: jumping repeatedly for 10 seconds with high effort level, bringing knees up as high as possible so both thighs were parallel with the ground, landing softly in the same footprint (2 pieces of tape) with each jump, and then immediately begin the next jump. No feedback was given to participants while performing the assessment. After 10 seconds, participants ceased jumping and cameras stopped recording.

**Figure 2:**
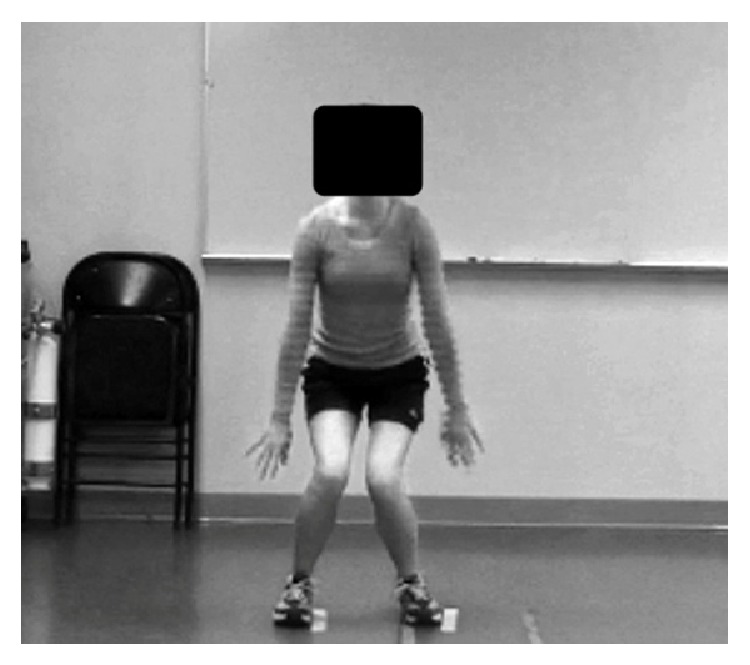
Lower extremity valgus at landing.

Raters used a previously published form to score technique flaws [8]. Technique flaws included: (1) lower extremity valgus at landing (Figure 2), (2) thighs do not reach parallel (peak of jump), (3) thighs not equal side-to-side (during flight), (4) foot placement not shoulder width apart, (5) foot placement not parallel (front to back), (6) foot contact timing not equal, (7) excessive landing contact noise, (8) pause between jumps, (9) technique declines prior to 10 seconds, and (10) does not land in same footprint (excessive in-flight motion) [8]. Additional figures depicting these technique flaws can be found in previously published TJA studies [6, 8, 10]. The participants were rated as either demonstrating a technique flaw or not. Per previously published literature, the flaws were then summed for the TJA total score [6, 8].

### 2.3. Raters

Five raters of varying educational backgrounds and clinical experiences were chosen to analyze video and score the TJA of the 40 participants. Raters included a physical therapist with a doctor of physical therapy degree and 4 years clinical experience (rater 1); the head strength and conditioning coach at a Division 1 university with a Masters of Science in Exercise Science and Strength and Conditioning Specialist certification and 7 years of clinical experience (rater 2); the head athletic trainer as a certified athletic trainer at a Division 1 university with 17 years of clinical experience (rater 3); a third-year doctor of physical therapy student (rater 4); and a first-year doctor of physical therapy student (rater 5).

All raters were given identical flash drives with instructions, a Microsoft Access database to input scores, a copy of Myer et al. [6] that described the TJA and scoring in detail, 80 video files for 40 participants (frontal and sagittal plane view for each participant), and 2 video files for 1 example participant (frontal and sagittal plane views) previously scored independently by 3 of the authors of this paper (L. A. Dudley, C. A. Smith, and M. Warren). The sample video was chosen from the larger sample of 100 to assist with consistency training of the raters and was not included in the 40 participants evaluated by raters. Raters were instructed to read an excerpt from Myer et al. [6] to create consistency between raters regarding the scoring procedures of the TJA. This included looking at the TJA scoring tool created with pictures of the first 6 technique flaws to ensure consistency with previously established TJA scoring procedures. Raters were then instructed to watch and score the example video, which had the purpose of ensuring consistency in procedures between all raters and establishing the same volume setting to accurately analyze technique flaw number 7 (excessive contact landing noise).

Once raters accurately scored the example video, participants' videos were scored independently, with no discussion amongst raters. Raters were instructed to use frontal plane views to score technique flaws (1), (3), (4), (6), and (7); sagittal plane views for (2) and (5); and frontal and sagittal views for (8), (9), and (10). Raters were given instructions on how to change playback speed on the computer program to allow viewing of the video in slow motion. Raters were encouraged to watch the videos as many times as necessary to give an accurate score. Approximately 1 month later, 3 of the 5 raters scored the videos again to determine intrarater reliability.

### 2.4. Statistical Analysis

Descriptive statistics were calculated as means with standard deviation for interval data and percents for categorical data. Analysis of variance with TJA total score as the dependent variable and rater as the independent variable was completed to assess differences in mean values between raters. Post hoc comparisons were calculated to compare means scores between each rater. Intraclass correlation coefficients (ICC, 2,1) and associated 95% confidence intervals (CI) were calculated to determine intrarater (3 different raters at 2 timepoints) and interrater (5 raters for first session and 3 raters for second session) reliability of the total TJA score. The clinical significance was defined as poor for an ICC below 0.50, moderate for 0.50–0.75, and good for 0.75 or higher [13]. Methods for calculation were adapted from Hankinson et al. [14] ICC was calculated as

(1) $$\begin{array}{c} \text{ICC}=\frac{{\sigma^{2}}_{B}}{{\sigma^{2}}_{B}+{\sigma^{2}}_{W}}, \end{array}$$

where *σ* ^2^_*B*_ is the between person variance and *σ* ^2^_*W*_ in the within person variance. Maximum likelihood estimates of *σ* ^2^_*B*_ and *σ* ^2^_*W*_ were obtained from linear mixed models with rater as a fixed effect for interrater reliability. Least square means were calculated for the mean TJA scores for each rater, with *P* values calculated to determine significant differences in TJA scores. All analyses were completed using SAS, Version 9.2 (SAS Institute, Inc., Cary, NC) and an a priori alpha level of 0.05 was used to denote statistical significance.

## 3. Results

Participants in this study were 40 university students, 18 to 24 years of age (mean age ± standard deviation (SD) 21.0 ± 1.6 years). There were 13 males and 27 females with an average height and weight of 170.8 ± 8.9 cm and 67.4 ± 14.7 kg, respectively. The average number of flaws identified on the TJA (TJA total score) were 6.30 ± 1.76; and technique flaw (2) (thighs do not reach parallel) was the most frequently identified (87.5% of participants), using scoring from rater 5 who was randomly chosen. Additionally, technique flaw (2) was the most consistently agreed upon by all raters. Technique flaws (5) (foot placement not parallel front to back) and (9) (technique declines prior to 10 seconds) were the least agreed upon by all raters.

Analysis of variance showed differences between raters for mean TJA total score (*F* = 11.82; *P* < 0.001). Post hoc comparisons showed no consistent pattern in scoring by educational and/or clinical experience (Table 1).

Interrater reliability between the 5 raters was poor (ICC = 0.47; 95% CI: 0.33–0.62). However, interrater reliability between raters 1, 2, and 5 (raters used for intrarater reliability) improved in the second scoring session (ICC = 0.52, 95% CI: 0.35–0.68 versus ICC = 0.69, 95% CI: 0.55–0.81).

Intrarater reliability testing was completed by raters 1, 2, and 5 who scored the TJA videos at two different timepoints. Intrarater reliability was poor to moderate (Table 2).

## 4. Discussion

The purpose of this study was to investigate intrarater and interrater reliability of the TJA with raters of different educational backgrounds and levels of clinical experience with healthy injury-free men and women. This study showed intrarater reliability to be poor to moderate for the 3 raters scoring videos of 40 participants. Each rater had a different educational background and level of experience. It is noteworthy that those with more education and experience were not more consistent in scoring the TJA than those raters with minimal education and experience. Rater 5 had highest intrarater reliability of the 3 raters and also had the most experience administering the TJA. Rater 5's experience may have contributed to higher consistency in scoring the TJA. These results suggest the TJA may not be used reliably, following published protocol, by a single clinician regardless of level of education or experience [6, 8, 11].

This study also showed poor interrater reliability between 5 raters when assessing TJA performance of the same 40 participants. This suggests the TJA may not be used reliably, following published protocol, when being scored by different raters [6, 8, 11]. However, interrater reliability improved the second session between the 3 raters that scored the videos in 2 separate sessions. Additional training beyond what was done in the study, as well as practice scoring to achieve consensus between raters, may be required.

These results are in contrast to Herrington et al. [11] who reported the TJA had good intrarater and interrater reliability (greater than 0.75) when 2 raters scored videos of 10 recreationally active university students at 2 different scoring sessions. There are several possibilities for the differences in reliability. Herrington et al. [11] did not specify raters' educational background, level of experience, or previous training in the TJA. However, as this study was written by a developer of the TJA, the two raters likely had more experience in scoring the TJA and were more familiar with the flaws than the 5 raters of this study. The improved interrater reliability in the current study from session one to session two suggested a possible learning effect. Further, the small sample size used by Herrington et al. [11] could promote bias due to raters remembering the scores of only 10 participants.

The authors of the current study took measures to ensure continuity with previously established TJA procedures and scoring criteria. However, current TJA instructions do not specify whether a technique flaw should be scored only if it is seen consistently throughout the test or if the technique flaw may be scored if seen only once during the test. Due to this exclusion in the published protocol, it is likely that raters may inherently interpret this differently, thus creating scoring inconsistencies between raters. For example, during a 10 second TJA a participant may demonstrate lower extremity valgus at landing only once throughout several jumps. One rater may score this as a technique flaw because it was demonstrated in the assessment; however, another rater may not score this as a technique flaw because the majority of jumps did not demonstrate lower extremity valgus at landing. More training and specific TJA instruction prior to administration may correct this issue and promote consistency between raters.

The current study includes the most diverse set of raters to date, representing 3 professions that may administer the TJA. This allows for greater external validity in the clinical and performance setting than other published reliability studies to date [11].

However, there are limitations of the current study. The TJA was developed to detect technique flaws in athletes [8]. Participants in the current study were not competitive athletes, though were recreationally active university students.

The results of this study suggest the TJA may not be consistently scored, following the previously published protocol, although modifications to instructions and rater training may correct this. Since, interrater reliability improved with repetition, more training in TJA administration may improve reliability. Further research is necessary to determine whether more specific scoring guidelines or training in TJA administration would improve reliability.

## 5. Conclusion

Using a published protocol and training of raters, the TJA has poor to moderate interrater and intrarater reliability. There may be a learned effect with the TJA since interrater reliability improved with repetition. Enhanced training or scoring instructions may be required to improve reliability of the TJA among professionals of varying education backgrounds and experience.

## Figures and Tables

**Table 1:** Mean TJA total score by rater.

Rater	Rater description	Mean ± standard deviation (total score)
1	Doctor of physical therapy with 4 years of experience^a^	6.86 ± 1.71
2	Master of science in exercise science with 7 years of experience^b^	6.03 ± 1.97
3	Certified athletic trainer with 17 years of experience^c^	4.65 ± 2.03
4	Third-year doctor of physical therapy student^c^	4.70 ± 1.64
5	First-year doctor of physical therapy student^a,b^	6.30 ± 1.76

Superscript letters signify statistically significant differences (*P* < 0.05) between raters. For example, rater 1 is not statistical different from number 5 but is significantly different from numbers 2, 3, and 4.

**Table 2:** Intrarater reliability ICC and 95% CI for each rater of TJA.

Rater number	Rater description	Intraclass correlation coefficient (ICC)	95% confidence interval (CI)
1	Doctor of physical therapy with 4 years of experience	0.57	0.36–0.76
2	Master of science in exercise science with 7 years of experience	0.44	0.22–0.68
5	First-year doctor of physical therapy student	0.72	0.55–0.84

^*^TJA: Tuck jump assessment.
